# Solubility, Hansen Solubility Parameters and Thermodynamic Behavior of Emtricitabine in Various (Polyethylene Glycol-400 + Water) Mixtures: Computational Modeling and Thermodynamics

**DOI:** 10.3390/molecules25071559

**Published:** 2020-03-28

**Authors:** Faiyaz Shakeel, Nazrul Haq, Ibrahim A. Alsarra, Sultan Alshehri

**Affiliations:** Department of Pharmaceutics, College of Pharmacy, King Saud University, P.O. Box 2457, Riyadh 11451, Saudi Arabia; fsahmad@ksu.edu.sa (F.S.); nhaq@ksu.edu.sa (N.H.); ialsarra@ksu.edu.sa (I.A.A.)

**Keywords:** computational modeling, cosolvent, emtricitabine, solubility, solubility parameter, thermodynamics

## Abstract

This study was aimed to find out the solubility, thermodynamic behavior, Hansen solubility parameters and molecular interactions of an antiviral drug emtricitabine (ECT) in various “[polyethylene glycol-400 (PEG-400) + water]” mixtures. The solubility of ECT in mole fraction was determined at “*T* = 298.2 to 318.2 K” and “*p* = 0.1 MPa” using an isothermal method. The experimental solubilities of ECT in mole fraction were validated and correlated using various computational models which includes “Van’t Hoff, Apelblat, Yalkowsky-Roseman, Jouyban-Acree and Jouyban-Acree-Van’t Hoff models”. All the models performed well in terms of model correlation. The solubility of ECT was increased with the raise in temperature in all “PEG-400 + water” mixtures studied. The highest and lowest solubility values of ECT were found in pure PEG-400 (1.45 × 10^−1^) at “*T* = 318.2 K” and pure water (7.95 × 10^−3^) at “*T* = 298.2 K”, respectively. The quantitative values of activity coefficients indicated higher interactions at molecular level in ECT and PEG-400 combination compared with ECT and water combination. “Apparent thermodynamic analysis” showed an “endothermic and entropy-driven dissolution” of ECT in all “PEG-400 + water” combinations studied. The solvation nature of ECT was found an “enthalpy-driven” in each “PEG-400 + water” mixture studied.

## 1. Introduction

**Figure 1:**
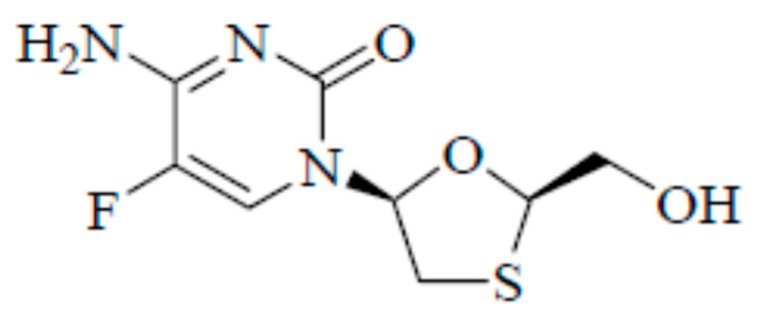
Chemical structure of emtricitabine (ECT).

Chemically, emtricitabine (ECT) is 5-fluoro-1-(2*R*,5*S*)-[2-(hydroxymethyl)-1,3-oxathiolan-5-yl]cytosine (Figure 1) [1]. Its molecular formula, molar mass and CAS registry number are C_8_H_10_FN_3_O_3_S, 247.24 g mol^−1^ and 143491-54-7, respectively [1,2].

It is a potential inhibitor of human immunodeficiency virus type I (HIV-I) reverse transcriptase and, hence, found to be effective against HIV-I infected patients [1,3]. ECT is prescribed to treat HIV-I infected patients either alone or in combination with other antiviral agents [3,4,5]. ECT is marketed as Emtriva^®^ in the treatment of HIV-I infected patients [1,5]. It has been found freely soluble in water at room temperature and, hence, its oral solution is available in the market [1]. It is extensively and rapidly absorbed by oral administration of capsules or oral solution due to its higher aqueous solubility [5]. The solubilities and other physicochemical parameters of ECT are poorly reported in literature. The solubility profiles and physicochemical properties of drugs and pharmaceuticals in cosolvent–water mixtures have greater impact for drug discovery process and formulation design [6,7,8]. Therefore, such physicochemical properties of ECT in cosolvent–water mixtures should be evaluated properly in order to obtain its complete physicochemical profile [6,7]. “Polyethylene glycol-400 (PEG-400)” has been reported as an efficient cosolvent in solubilization of drugs in aqueous media and it is miscible with water in all proportions [9,10,11]. The potential of PEG-400 has been studied extensively in solubility enhancement of various drugs [9,10,11,12,13,14,15,16,17,18]. Several formulation approaches including “3D printed controlled release tablets [4], film coated tablets [19], rapidly disintegrating vaginal tablets [20], immediate release tablets [21], vaginal gels [22], liposomal gels [23], microparticulate drug delivery system [24], nanosuspensions [25,26] and polymeric nanoparticles [5,27,28]” were reported to improve antiviral therapy and pharmacokinetic profile of ECT. The equilibrium solubility of ECT in water was found as 112 mg mL^−1^ at “*T* = 298.2 K” [1]. Nevertheless, the solubilities of ECT in different “PEG-400 + water” mixtures have not been reported in literature so far. Hence, this study was aimed to find out the solubility, Hansen solubility parameters, solution thermodynamics and solute–solvent molecular interactions of ECT in various “PEG-400 + water” mixtures, including mono solvents at “*T* = 298.2 to 318.2 K” and “*p* = 0.1 MPa”. The effect of pressure on ECT solubility was not evaluated in the current work and; therefore, current work was performed at fixed air pressure (i.e., “*p* = 0.1 MPa”). The investigated temperature ranges of “*T* = 298.15 K to 318.15 K” were selected randomly with the interval of 5.0 K in such a manner that the highest studied temperature (i.e., “*T* = 318.2 K”) should not exceed the fusion temperature of ECT and boiling points of the investigated solvents (i.e., water and PEG-400 in this case) [6,29]. The fusion temperature of ECT was obtained as 427.80 K by thermal analysis. The boiling temperatures of water and PEG-400 are 373.20 and 563.20 K, respectively. The highest studied temperature “T = 318.2 K” was much lower than fusion temperature of ECT and boiling temperatures of water and PEG-400 and therefore the above temperature range was chosen in the current research. Experimental solubility values of ECT were correlated with five different computational models namely “Van’t Hoff, Apelblat, Yalkowsky-Roseman, Jouyban-Acree and Jouyban-Acree-Van’t Hoff models”. The solubility data and other physicochemical properties of ECT recorded in this study would be beneficial in “drug discovery process and dosage form design of ECT”.

## 2. Results and Discussion

### 2.1. Solid State Characterization of ECT

**Figure 2:**
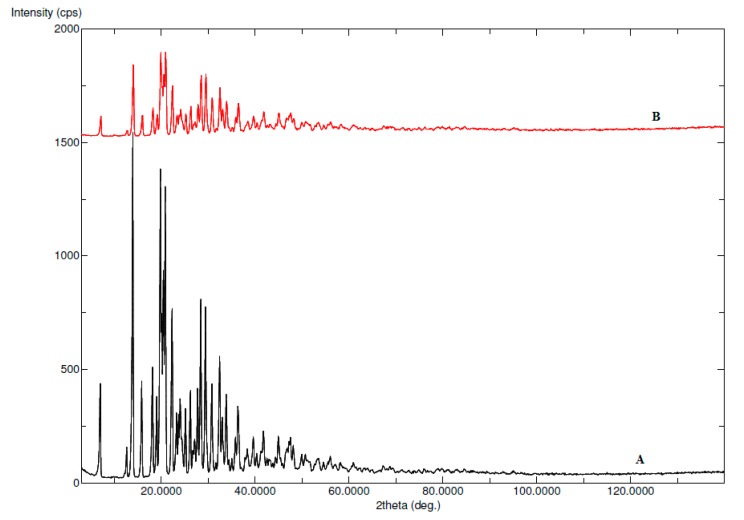
X-ray diffraction pattern (XRD) spectra of (**A**) pure ECT and (**B**) equilibrated ECT (recovered from pure water).

The solid state characterization of ECT in pure and equilibrated sample was conducted using X-ray diffraction (XRD) analysis. The XRD patterns of pure and equilibrated ECT are presented in Figure 2. The XRD patterns of pure ECT showed various characteristics peaks of ECT at 2θ = 6.70°, 12.40°, 13.70°, 15.60°, 17.90°, 19.60°, 20.60°, 22.10°, 24.00°, 26.00°, 29.30°, 30.60° and 32.20° (Figure 2A). The characteristics peaks at various 2θ values suggested the crystallinity of the pure ECT. The XRD patterns of equilibrated ECT (recovered from pure water) also showed almost the same characteristic peaks at various 2θ values (Figure 2B). The results of XRD analysis also showed that ECT was not transformed into polymorphs/hydrates/solvates after saturation. Overall, the XRD spectra of pure and equilibrated ECT suggested that the physical form of the ECT remained unchanged after solubility experiments [29].

### 2.2. Solubility Data of ECT in Various “PEG-400 + Water” Mixtures

The experimental mole fraction solubilities of ECT (*x*_e_) in different “PEG-400 + water” combinations and mono solvents at “*T* = 298.2 to 318.2 K” and “*p* = 0.1 MPa” were calculated using Equations (1) and (2) and results are summarized in Table 1.

**Figure 3:**
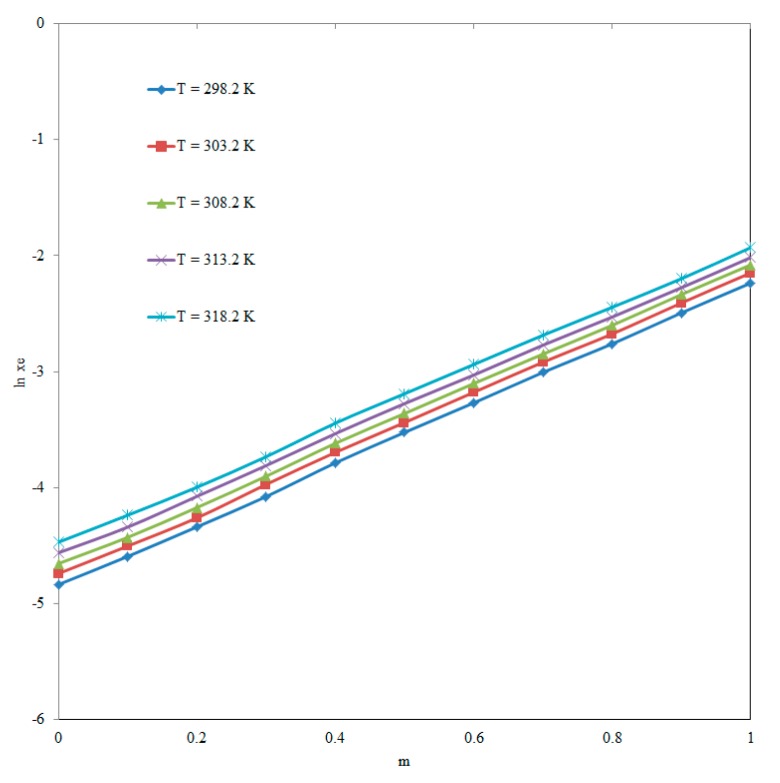
Effect of mass fraction (*m*) value of the PEG-400 on the solubility of ECT at “*T* = 298.2 to 318.2 K”.

The solubility of ECT in various “PEG-400 + water” combinations including mono solvents at different temperatures is not reported elsewhere. However, the equilibrium solubility of ECT in pure water at “*T* = 298.2 K” has been reported as 112 mg mL^−1^ (converted to 8.09 × 10^−3^ in mole fraction) [1]. The mole fraction solubility of ECT in pure water at “*T* = 298.2 K” was obtained as 7.95 × 10^−3^ in this study (Table 1). The recorded solubility of ECT in pure water “*T* = 298.2 K” was very close to its literature value [1]. The reliability of the method was verified by determining the mole fraction solubility of isatin in pure water at *T* = 298.2 K and *T* = 318.2 K. The mole fraction solubility of isatin in pure water at *T* = 298.2 K and *T* = 318.2 K has been reported as 5.14 × 10^−5^ and 1.13 × 10^−4^, respectively [11]. The mole fraction solubility of isatin in water at *T* = 298.2 K and *T* = 318.2 K was found as 5.12 × 10^−5^ and 1.16 × 10^−4^, respectively in this study. These results showed that the solubility of isatin in water recorded using the present method was much closed with its literature values [11]. Hence, the current method of solubility determination was accurate and reliable for the determination of ECT solubility. In general, the *x*_e_ values of ECT were found to enhance linearly with increase in temperature in all “PEG-400 + water” combinations and the *x*_e_ value of ECT was increased as the fraction of PEG-400 in “PEG-400 + water” mixtures increased (*p* < 0.05). Highest *x*_e_ of ECT was recorded in pure PEG-400 (1.45 × 10^−1^ at “*T* = 318.2 K”), whereas, the lowest one was observed in pure water (7.95 × 10^−3^ at “*T* = 298.2 K”). Highest *x*_e_ of ECT in pure PEG-400 could be possible due to lower polarity and lower Hansen solubility parameter (HSP) of PEG-400 in comparison with same physicochemical parameters of water [7,29]. The impact of mass fraction value of PEG-400 (*m*) on the solubility of ECT at “*T* = 298.2 to 318.2 K” was also investigated and resulting data is summarized in Figure 3. The results indicated significant increase in the solubility of ECT with increase in *m* value of PEG-400 in “PEG-400 + water” mixtures at “*T* = 298.2 to 318.2 K” (*p* < 0.05). In this study the influence of molar mass of PEGs on the solubility of ECT was not studied because only PEG-400 (average molar mass = 400 g mol^−1^) was studied in this work. Nevertheless, it is well known that the solubility of solute in mole fraction is enhanced with increase in the molar mass of the solutes and the solvents. Hence, in this case, the solubility of ECT in mole fraction would be increased with increase in the molar mass of PEGs [30]. This observation is possible due to the fact that the solubility of ECT in mole fraction is inversely proportional to the mole fraction of the PEGs. It was also found that the solubility of ECT enhanced significantly from pure water to pure PEG-400 (*p* < 0.05). Therefore, PEG-400 can be applied as a physiologically compatible cosolvent in solubilization of ECT in water. Overall, the solubility of ECT in pure water, pure PEG-400 and various “PEG-400 + water” combinations was found good.

### 2.3. HSPs for ECT and Various “PEG-400 + Water” Mixtures

The HSPs for ECT, pure PEG-400 and pure water were calculated using “HSPiP software”. The HSPs of ECT, pure PEG-400 and pure water were calculated using Equation (3). However, the HSP of various “PEG-400 + water” mixtures free of ECT was calculated using Equation (4). The total HSP for ECT (*δ*) was calculated as 25.90 MPa^1/2^. The value of total HSP for pure PEG-400 (*δ*_1_) and pure water (*δ*_2_) were calculated as 18.90 and 47.80 MPa^1/2^, respectively. The value of HSP for various “PEG-400 + water” mixtures free of ECT (*δ*_mix_) was estimated by applying Equation (4) and these values are presented in Table 2.

The values of *δ*_mix_ for various “PEG-400 + water” combinations were found as 21.79 to 44.91 MPa^1/2^. Overall, the HSP of pure PEG-400 (*δ*_1_ = 18.90 MPa^1/2^) and “PEG-400 + water” (at *m* = 0.6 to 0.9; *δ*_mix_ = 21.79 to 30.46 MPa^1/2^) were found to have close value with that of ECT (*δ* = 25.90 MPa^1/2^). Overall, the results of HSPs were found in good agreement with experimental solubility values of ECT in various “PEG-400 + water” combinations.

### 2.4. Ideal Solubilities and Activity Coefficients

The ideal solubility (*x*^idl^) of ECT at “*T* = 298.2 to 318.2 K” was obtained by applying Equation (5) and resulting values are summarized in Table 1. The *x*^idl^ values of ECT were observed as 3.74 × 10^−2^ to 6.76 × 10^−2^ at “*T* = 298.2 to 318.2 K”. The *x*^idl^ values of ECT were significantly higher than its *x*_e_ values in pure water (*p* < 0.05). However, the *x*^idl^ values of ECT were significantly lower than its *x*_e_ values in pure PEG-400 (*p* < 0.05) at each temperature evaluated. While, the *x*^idl^ values of ECT were more closed with its *x*_e_ values in various “PEG-400 + water” mixtures (*p* ˃ 0.05). Due to maximum solubility of ECT in PEG-400, it could be applied as “an ideal cosolvent” for solubilization of ECT.

The “activity coefficient (*γ*_i_)” for ECT in various “PEG-400 + water” mixtures at “*T* = 298.2 to 318.2 K” were estimated by applying Equation (6) and resulting data is summarized in Table 3. The *γ*_i_ values for ECT were observed highest in pure water compared with pure PEG-400 and various “PEG-400 + water” mixtures at every temperature evaluated. While, the *γ*_i_ values for ECT were found lowest in pure PEG-400 at every temperature evaluated. The *γ*_i_ values were found <1.0 at *m* = 0.6 to 0.9 and pure PEG-400. The *γ*_i_ values for ECT were found to reduce rapidly from pure water to pure PEG-400 (*p* < 0.05). The highest *γ*_i_ of ECT in pure water could be possible due to the lowest solubility of ECT in water and higher dielectric constant/polarity of water compared with the highest solubility of ECT in PEG-400 and lower dielectric constant/polarity of neat PEG-400 [15,17]. The results of activity coefficients for ECT in various “PEG-400 + water” mixtures were found in accordance with their dielectric constants and mole fraction solubility [15]. Based on these results, the maximum solute–solvent interactions were found in ECT-PEG-400 compared with ECT-water. 

### 2.5. Thermodynamic Parameters of ECT

The values of various apparent thermodynamic parameters for dissolution behavior of ECT in different “PEG-400 + water” mixtures and pure solvents were obtained by applying van’t Hoff and Gibbs Equations (7–10) and results are summarized in Table 4. The values of apparent standard enthalpies (*Δ*_sol_*H*^0^) for ECT dissolution in various “PEG-400 + water” mixtures and pure solvents were found as positive values in the range of 11.75 to 14.35 kJ mol^−1^, indicating “endothermic dissolution” of ECT in all cosolvent mixtures including pure water and pure PEG-400 [6,7]. The “*Δ*_sol_*H*^0^ values” of ECT were found to decrease with increase in the *m* value of PEG-400 in “PEG-400 + water” mixtures and solubility of ECT. Hence, the maximum “*Δ*_sol_*H*^0^ value” was found in pure water (14.35 kJ mol^−1^), while, the minimum one was recorded in pure PEG-400 (11.75 kJ mol^−1^). 

The values of apparent standard Gibbs free energies (*Δ*_sol_*G*^0^) for ECT dissolution in various “PEG-400 + water” mixtures were also recorded as positive values in the range of 5.32 to 11.91 kJ mol^−1^ (Table 4). The “*Δ*_sol_*G*^0^ values” for ECT dissolution were also found decreased with increase in *m* value of PEG-400 in “PEG-400 + water” mixtures and solubility values of ECT. The maximum and minimum “*Δ*_sol_*G*^0^ values” for ECT dissolution were observed in pure water (11.91 kJ mol^−1^) and pure PEG-400 (5.32 kJ mol^−1^), respectively.

The values of apparent standard entropies (Δ_sol_*S*^0^) for ECT dissolution in various “PEG-400 + water” mixtures were also obtained as positive values in the range of 9.94 to 20.86 J mol^−1^ K^−1^, indicating “entropy-driven dissolution” of ECT in all “PEG-400 + water” combinations including pure water and pure PEG-400 [7]. The mean relative uncertainties in “*Δ*_sol_*H*^0^, *Δ*_sol_*G*^0^ and *Δ*_sol_*S*^0^” were found as 0.07, 0.25 and 0.30, respectively. Based on these results, the overall dissolution of ECT was found as an “endothermic and entropy-driven” in all “PEG-400 + water” mixtures and pure solvents [6,7].

### 2.6. Solvation Analysis of ECT

**Figure 4:**
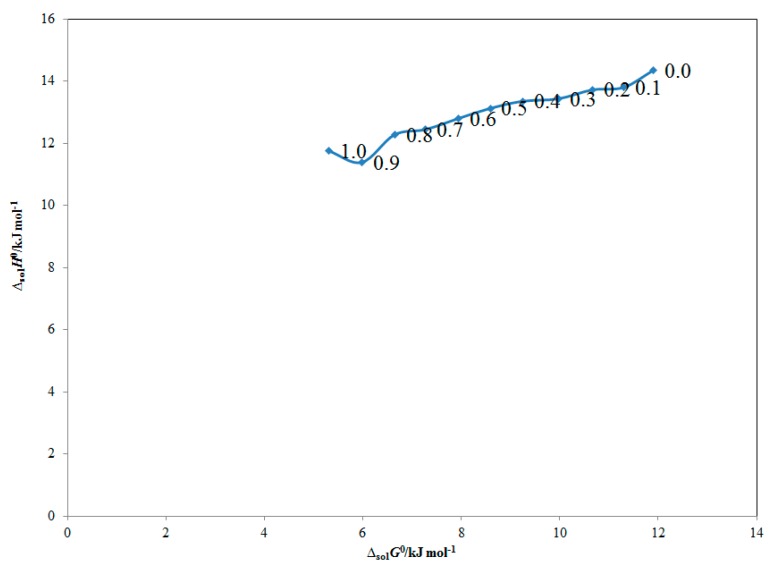
Apparent standard enthalpy (*Δ*_sol_*H*^0^) vs. apparent standard Gibbs energy (*Δ*_sol_*G*^0^) “enthalpy–entropy compensation” graph for ECT in different “PEG-400 + water” combinations at *T*_hm_ value of 308 K.

The solvation behavior of ECT in various “PEG-400 + water” mixtures was studied using “enthalpy–entropy compensation analysis” and results are summarized in Figure 4. It was found that ECT in all “PEG-400 + water” mixtures and pure solvents expressed a non-linear “Δ_sol_*H*° vs. Δ_sol_*G*°” curve with a positive slope value of 1.40. Based on this observation, the “driving mechanism” for ECT solvation was proposed as an “enthalpy-driven” in all “PEG-400 + water” mixtures and pure solvents. This observation was probably due to higher solvation of ECT in pure PEG-400 molecules compared with pure water molecules [7]. The solvation behavior of ECT in various “PEG-400 + water” mixtures recorded in this study was in accordance with those proposed for the solvation mechanism of ferulic acid, 4-(4-ethoxyphenyl)-5-(3,4,5-trimethoxybenzoyl)-3,4-dihydropyrimidine-2(1*H*)-one, lornoxicam, hydrazide derivative, isatin, paracetamol and sulfadiazine in various “PEG-400 + water” mixtures [7,11,12,31,32,33,34].

### 2.7. Computation Modeling

The experimental solubility data of ECT was correlated and validated using five different computational models including “Van’t Hoff, Apelblat, Yalkowsky-Roseman, Jouyban-Acree and Jouyban-Acree-Van’t Hoff models”. The “Van’t Hoff model solubility (*x*^Van’t^)” of ECT was calculated by applying Equation (11) and the *x*_e_ value of ECT was correlated with its *x*^Van’t^ value using “root mean square deviation (*RMSD*) and determination coefficient (*R*^2^)” values. The resulting data of “Van’t Hoff model” for ECT in various “PEG-400 + water” mixtures, including pure water and pure PEG-400, are summarized in Table 5. *RMSD*s for ECT in various “PEG-400 + water” mixtures, including pure water and pure PEG-400, were computed as 0.42% to 0.91% with an overall *RMSD* value of 0.73%. The *R*^2^ values for “Van’t Hoff model” were obtained as 0.9955 to 0.9995.

The “Apelblat model solubility (*x*^Apl^)” of ECT was calculated by applying Equation (12) and the *x*_e_ value of ECT was correlated with its *x*^Apl^ value in using “*RMSD* and *R*^2^” values. The results of the “Apelblat model” for ECT in various “PEG-400 + water” mixtures and pure solvents are summarized in Table 6.

**Figure 5:**
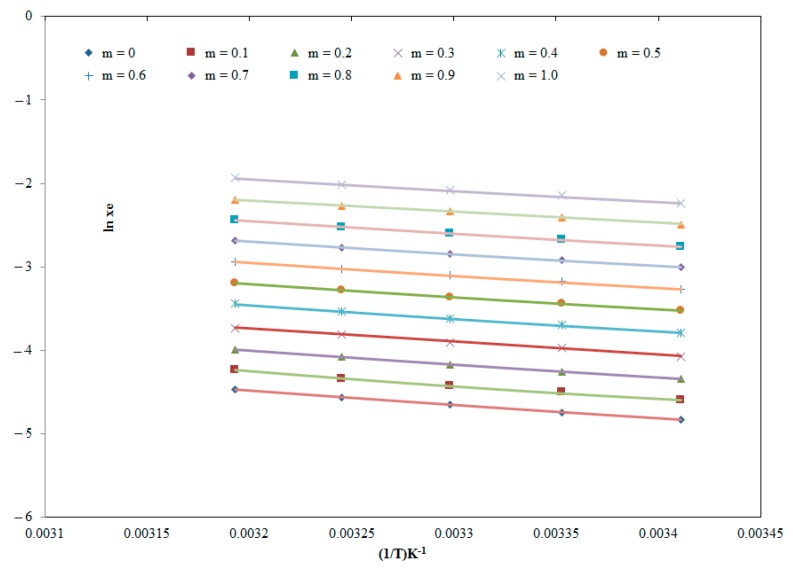
Graphical correlation of logarithmic solubilities of ECT with the “Apelblat model” in various “PEG-400 + water” combinations at “*T* = 298.2 to 318.2 K” (solid lines indicate the “Apelblat solubilities” of ECT and symbols indicate the experimental solubilities of ECT).

The *RMSD* values for ECT in various “PEG-400 + water” mixtures and pure solvents were estimated as 0.19% to 0.74% with an overall *RMSD* of 0.46%. The *R*^2^ values for the “Apelblat model” were recorded as 0.9968 to 0.9998. Graphical correlation between *x*_e_ and *x*^Apl^ values of ECT are summarized in Figure 5, indicating good graphical correlation of *x*_e_ values of ECT with the “Apelblat model”.

The “logarithmic solubility of Yalkowsky-Roseman model (log *x*^Yal^)” of ECT was calculated by applying Equation (13) and the correlation between *x*_e_ and *x*^Yal^ values of ECT was performed in terms of *RMSD* values.

Resulting data of the “Yalkowsky-Roseman model” for ECT in various “PEG−400 + water” mixtures, including pure water and pure PEG-400, are summarized in Table 7. The *RMSD*s for the “Yalkowsky-Roseman model” were found as 0.44% to 2.98% with an overall *RMSD* value of 1.33%.

Resulting data of the “Jouyban-Acree model” (Equation (14)) and the “Jouyban-Acree-Van’t Hoff model” (Equation (15)) for ECT in “PEG-400 + water” mixtures are summarized in Table 8. An overall *RMSD* for the “Jouyban-Acree model” was found as 0.42%. While, an overall *RMSD* for the “Jouyban-Acree-Vant’t Hoff model” was found as 0.61%. In general, all computational models represented good correlation based on *RMSD* values, but the “Jouyban-Acree” model was found as the most accurate and precise because it utilized the least number of model parameters compared with other models.

## 3. Materials and Methods

### 3.1. Materials

ECT was procured from “Sigma-Aldrich (St. Louis, MO, USA). PEG-400 (average molar mass = 400 g mol^−1^ and polydispersity index = 1.04) was obtained from Fluka Chemica (Busch, Switzerland). The chromatography-grade water was collected from “Milli-Q water purification unit” in the laboratory. The list of materials along with their detailed properties are summarized in Table 9.

### 3.2. Solid Phase Characterization of ECT

The solid phase characterization of ECT in its pure form and equilibrated sample was performed by XRD analysis. The XRD analysis of ECT in pure and equilibrated samples was performed by “Ultima IV Diffractometer (Rigaku Inc. Tokyo, Japan)”. The diffraction angle (2θ) range for this analysis was set at 3°−120° with a scan speed of 1.0° min^−1^ for both samples. The rest of the operation and condition was similar to those reported in our previous article [29].

### 3.3. Determination of ECT Solubility in Various “PEG-400 + Water” Mixtures

The solubility of ECT in various “PEG-400 + water” mixtures and mono solvents was determined at “*T* = 298.2 to 318.2 K” and “*p* = 0.1 MPa” using a reported isothermal method [35]. Before determining the solubility of ECT, the reliability of experimental method was verified by determining the solubility of isatin in pure water at *T* = 298.2 and *T* = 318.2 K. The solubility of isatin in pure water could be compared with existing literature values [11]. The excess amount of ECT was taken in fixed amounts of various “PEG-400 + water” mixtures and mono solvents. Each analysis was repeated for three times (*n* = 3). The obtained dispersions were vortex mixed for about 5 min and transferred to “WiseBath^®^ WSB Shaking Water Bath (Model WSB-18/30/-45, Daihan Scientific Co. Ltd., Seoul, Korea)” for equilibrium/saturation. The speed and equilibrium time of shaker were set at 100 rpm and 72 h, respectively [7]. The temperature of measurement was changed as mentioned above. The uncertainty in the temperature of “WiseBath^®^ WSB Shaking Water Bath” was computed as ± 0.10 K. After 72 h, each mixture was withdrawn carefully and ECT particles were allowed to settle overnight [7,8]. Then, the supernatant of each sample was taken, diluted (wherever applicable) and subjected for the analysis of ECT concentration by high-performance liquid chromatography method at the wavelength of 254 nm. The binary mixture of “methanol:ethanol (1:1 % v/v)” was used as a mobile phase for this analysis. The *x*_e_ values of ECT were calculated using the following Equations [32,33]:

(1) $$x_{e}=\frac{m_{1}/M_{1}}{m_{1}/M_{1}+m_{2}/M_{2}}$$

(2) $$x_{e}=\frac{m_{1}/M_{1}}{m_{1}/M_{1}+m_{2}/M_{2}+m_{3}/M_{3}}$$

where *m*_1_ = mass of ECT; *m*_2_ = mass of PEG-400; *m*_3_ = mass of water; *M*_1_ = molar mass of ECT; *M*_2_ = molar mass of PEG-400 and *M*_3_ = molar mass of water.

### 3.4. HSPs of ECT and Various “PEG-400 + Water” Mixtures

The HSP of solute is associated with its solubility in pure solvent or cosolvent–water mixtures. If the HSP of solute is closed with that of pure solvent or cosolvent–water mixtures, the solubility of solute will be higher in that pure solvent or cosolvent–water mixture [36]. Hence, HSP for ECT, pure PEG-400, pure water and various “PEG-400 + water” mixtures free of ECT were computed in this study. The *δ* values for ECT, pure PEG-400 and pure water were computed using the following Equation [37,38,39]:

(3) $$\delta^{2}=\delta_{d}^{2}+\delta_{p}^{2}+\delta_{h}^{2}$$

where “*δ* = total HSP; *δ*_d_ = dispersion HSP; *δ*_p_ = polar HSP and *δ*_h_ = hydrogen-bonded HSP”. The values of HSP for ECT and mono solvents were computed using “HSPiP software (version 4.1.07, Louisville, KY, USA)” by putting the smiles of ECT and mono solvents into the software [38]. The HSP for various “PEG-400 + water” mixtures free of ECT (*δ*_mix_) was computed using the following Equation [40,41]:

(4) $$\delta_{\mathrm{mix}}=\propto \delta_{1}+\left(1-\propto \right)\delta_{2}$$

where *α* = volume fraction of PEG-400 in “PEG-400 + water” mixtures; *δ*_1_ = HSP of pure PEG-400 and *δ*_2_ = HSP of pure water.

### 3.5. Ideal Solubilities and Activity Coefficients

The *x*^idl^ values of ECT at five different temperatures were computed using the following Equation [42]:

(5) $$\ln\;x^{\mathrm{idl}}=\frac{-\Delta H_{\mathrm{fus}}\left(T_{\mathrm{fus}}-T\right)}{RT_{\mathrm{fus}}T}+\left(\frac{\Delta C_{p}}{R}\right)[\frac{T_{\mathrm{fus}}-T}{T}+\ln\left(\frac{T}{T_{\mathrm{fus}}}\right)]\;$$

where *T* = absolute temperature; *T*_fus_ = fusion temperature of ECT; *R* = universal gas constant; ∆*H*_fus_ = molar fusion enthalpy of ECT and ∆*C*_p_ = difference in the molar heat capacity of solid state with that of liquid state of ECT [42,43]. The quantitative values of “*T*_fus_, ∆*H*_fus_ and ∆*C*_p_” for ECT were computed as 427.80 K, 32.37 kJ mol^−1^ and 75.66 J mol^−1^ K^−1^, respectively using thermal analysis. Now, the *x*^idl^ values for ECT were computed by applying Equation (5).

The *γ*_i_ values for ECT in various “PEG-400 + water” mixtures were computed using the following equation [42,44]:

(6) $$\gamma_{i}=\frac{x^{\mathrm{idl}}}{x_{e}}$$

Using the quantitative values of *γ*_i_, the molecular interactions between solute and the solvents were computed. 

### 3.6. Thermodynamic Behavior of ECT

The dissolution behavior of ECT in various “PEG-400 + water” mixtures and mono solvents was evaluated by applying “apparent thermodynamic analysis” using “Van’t Hoff and Gibbs Equations”. The “van’t Hoff Equation” was used to found out apparent thermodynamic parameters of ECT in various “PEG-400 + water” mixtures, which was obtained at mean harmonic temperature (*T*_hm_) of 308 K within the temperature range of “*T* = 298.2 to 318.2 K”, and is expressed using the following Equation [42,45]:

(7) (∂ln xe∂(1T−1Thm))P=−ΔsolH0R

By plotting ln *x*_e_ values of ECT against 1T−1Thm, the *Δ*_sol_*H*^0^ and *Δ*_sol_*G*^0^ values for ECT dissolution were computed from the slope and intercept, respectively, using the following Equations [46]:

(8) ΔsolH0=−R(∂ln xe∂(1T−1Thm))P

(9) $$\Delta_{\mathrm{sol}}G^{0}=-RT_{\mathrm{hm}}\times \mathrm{intercept}$$

Finally, the *Δ*_sol_*S*^0^ values for ECT dissolution in various “PEG-400 + water” mixtures and mono solvents were computed using the following Gibbs Equation [42,45,46]:

(10) $$\Delta_{\mathrm{sol}}S^{0}=\frac{\Delta_{\mathrm{sol}}H^{0}-\Delta_{\mathrm{sol}}G^{0}}{T_{\mathrm{hm}}}$$

### 3.7. Enthalpy–Entropy Compensation Analysis

The solvation analysis of ECT in various “PEG-400 + water” mixtures and mono solvents was computed by applying “enthalpy–entropy compensation analysis” [7,45]. This analysis was conducted by plotting the weighted plots of “Δ_sol_*H*° vs. Δ_sol_*G*°” at *T*_hm_ = 308 K [7].

### 3.8. Computational Models

In this study, the *x*_e_ values of ECT were validated and correlated with five different computational models such as “Van’t Hoff, Apelblat, Yalkowsky-Roseman, Jouyban-Acree and Jouyban-Acree-Van’t Hoff” models [31,47,48,49,50,51,52].

The *x*^Van’t^ values of ECT in various “PEG-400 + water” combinations including pure water and pure PEG-400 were calculated by applying the following Equation [31]:

(11) $$\ln\;x^{\mathrm{Va}n\text{′}t}=a+\frac{b}{T}$$

where “*a* and *b* = model parameters of Equation (11)” which were computed by drawing the plots between ln *x_e_* values of ECT and 1/*T*. 

The *x*^Apl^ values of ECT in various “PEG-400 + water” mixtures including pure water and pure PEG-400 were obtained by applying the following Equation [47,48]:

(12) $$\ln\;x^{\mathrm{Apl}}=A+\frac{B}{T}+C\ln\left(T\right)$$

where “*A, B* and *C* = model parameters Equation (12)” which were computed by applying “nonlinear multivariate regression analysis” of *x*_e_ values of ECT summarized in Table 1 [31]. 

The log *x*^Yal^ values for ECT in various “PEG-400 + water” combinations including pure water and pure PEG-400 were estimated by applying the following Equation [49]:

(13) $$\mathrm{Log}x^{\mathrm{Yal}}=m_{1}logx_{1}+m_{2}logx_{2}$$

where *x*_1_ = mole fraction solubility of ECT in pure PEG-400; *x*_2_ = mole fraction solubility of ECT in pure water; *m*_1_ = mass fraction of pure PEG-400 and *m*_2_ = mass fraction of pure water in the absence of ECT.

The “Jouyban-Acree model solubility (*x*_m,T_)” of ECT in “PEG-400 + water” mixtures was estimated by applying the following Equation [31,50,51,52]:

(14) $$\ln x_{m,T}=m_{1}\ln x_{1}+m_{2}\ln x_{2}+\left[m_{1}m_{2}\overset{2}{\underset{i=0}{\sum }}\frac{J_{i}}{T}{\left(m_{1}-m_{2}\right)}^{i}\right]$$

where “*J*_i_ = model parameter of Equation (14)” and it was computed from “no-intercept regression analysis” [31,53].

The “Jouyban-Acree-van’t Hoff” solubility of ECT in “PEG-400 + water” mixtures was estimated by applying the following Equation [31,54]:

(15) $$\ln\;x_{m,T}=m_{1}\left(A_{1}+\frac{B_{1}}{T}\right)+m_{2}\left(A_{2}+\frac{B_{2}}{T}\right)+\left[\frac{m_{1}m_{2}}{T}\;\overset{2}{\underset{i=0}{\sum }}J_{i}{\left(m_{1}-m_{2}\right)}^{i}\right]$$

where “*A_1_*, *B_1_*, *A_2_*, *B_2_* and *J_i_* = model parameters of Equation (15)”.

### 3.9. Statistical Analysis

Statistical analysis was done by applying the “Kruskal–Wallis test” followed by the Denn’s test using “GraphpadInstat software (San Diego, CA, USA)”. The *p* < 0.05 was considered as significant value.

## 4. Conclusions

This study aimed to find out solubility, HSPs and apparent thermodynamic parameters of ECT in various “PEG-400 + water” mixtures and pure solvents at “*T* = 298.2 to 318.2 K” and “*p* = 0.1 MPa”. Experimental solubilities of ECT were correlated well by “Van’t Hoff, Apelblat, Yalkowsky-Roseman, Jouyban-Acree and Jouyban-Acree-Van’t Hoff” models. In general, all computational models performed well in terms of *RMSD* values, but the Jouyban-Acree model was found as the most accurate and precise as it utilized the least number of model parameters. The solubilities of ECT were found to enhance with the raise in temperature and the increase in the *m* value of PEG-400 in all “PEG-400 + water” mixtures and pure solvents. The solubility results were in accordance with their HSPs and polarity. The data of activity coefficients indicated maximum molecular interactions in ECT-PEG-400 compared with ECT-water. “Apparent thermodynamic analysis” showed an “endothermic and entropy-driven” dissolution of ECT in various “PEG-400 + water” mixtures and pure solvents. “Enthalpy–entropy compensation” analysis indicated that the solvation behavior of ECT was “enthalpy-driven” in all “PEG-400 + water” mixtures and mono solvents.

## Figures and Tables

**Table 1:** Experimental solubilities (*x*_e_) of emtricitabine (ECT) in mole fraction in various “polyethylene glycol-400 (PEG-400) + water” mixtures (*m*) at “*T* = 298.2 to 318.2 K” and “*p* = 0.1 MPa” ^a^.

*m*	*x*_e_				
	*T* = 298.2 K	*T* = 303.2 K	*T* = 308.2 K	*T* = 313.2 K	*T* = 318.2 K
0.0	7.95 × 10^−3^	8.74 × 10^−3^	9.53 × 10^−3^	1.05 × 10^−2^	1.15 × 10^−2^
0.1	1.01 × 10^−2^	1.11 × 10^−2^	1.19 × 10^−2^	1.30 × 10^−2^	1.44 × 10^−2^
0.2	1.31 × 10^−2^	1.41 × 10^−2^	1.54 × 10^−2^	1.70 × 10^−2^	1.84 × 10^−2^
0.3	1.70 × 10^−2^	1.88 × 10^−2^	2.02 × 10^−2^	2.22 × 10^−2^	2.39 × 10^−2^
0.4	2.27 × 10^−2^	2.48 × 10^−2^	2.68 × 10^−2^	2.92 × 10^−2^	3.20 × 10^−2^
0.5	2.95 × 10^−2^	3.21 × 10^−2^	3.47 × 10^−2^	3.78 × 10^−2^	4.12 × 10^−2^
0.6	3.81 × 10^−2^	4.17 × 10^−2^	4.50 × 10^−2^	4.84 × 10^−2^	5.30 × 10^−2^
0.7	4.95 × 10^−2^	5.41 × 10^−2^	5.80 × 10^−2^	6.26 × 10^−2^	6.82 × 10^−2^
0.8	6.33 × 10^−2^	6.90 × 10^−2^	7.43 × 10^−2^	7.98 × 10^−2^	8.68 × 10^−2^
0.9	8.28 × 10^−2^	9.01 × 10^−2^	9.69 × 10^−2^	1.03 × 10^−1^	1.11 × 10^−1^
1.0	1.06 × 10^−1^	1.16 × 10^−1^	1.25 × 10^−1^	1.33 × 10^−1^	1.45 × 10^−1^
*x* ^idl^	3.74 × 10^−2^	4.35 × 10^−2^	5.05 × 10^−2^	5.85 × 10^−2^	6.76 × 10^−2^

^a^ The standard uncertainties *u* are *u*(*T*) = 0.13 K, *u*_r_(*m*) = 0.10 %, *u*(*p*) = 0.003 MPa and *u*_r_(*x*_e_) = 1.10 %.

**Table 2:** Hansen solubility parameters (*δ*_mix_/MPa^1/2^) for different “PEG-400 + water” combinations free of ECT at “*T* = 298.2 K”.

*m*	*δ*_mix_/MPa^1/2^
0.1	44.91
0.2	42.02
0.3	39.13
0.4	36.24
0.5	33.35
0.6	30.46
0.7	27.57
0.8	24.68
0.9	21.79

**Table 3:** Activity coefficients (*γ_i_*) of ECT in various “PEG-400 + water” mixtures (*m*) at “*T* = 298.2 to 318.2 K”.

*m*	*γ*_i_				
	*T* = 298.2 K	*T* = 303.2 K	*T* = 308.2 K	*T* = 313.2 K	*T* = 318.2 K
0.0	4.71	4.99	5.31	5.60	5.91
0.1	3.70	3.94	4.24	4.49	4.68
0.2	2.87	3.09	3.28	3.44	3.68
0.3	2.21	2.31	2.50	2.64	2.83
0.4	1.65	1.75	1.88	2.01	2.12
0.5	1.27	1.36	1.46	1.55	1.64
0.6	0.98	1.05	1.12	1.21	1.28
0.7	0.75	0.80	0.87	0.93	0.99
0.8	0.59	0.63	0.68	0.73	0.77
0.9	0.45	0.48	0.52	0.57	0.60
1.0	0.35	0.37	0.40	0.43	0.46

**Table 4:** Apparent thermodynamic parameters [apparent standard enthalpy (Δ_sol_*H*^0^), apparent standard Gibbs energy (Δ_sol_*G*^0^) and apparent standard entropy (Δ_sol_*S*^0^)] and *R*^2^ values for ECT dissolution in various “PEG-400 + water” mixtures (*m*) ^b^.

*m*	Δ_sol_*H*^0^/kJ mol^−1^	Δ_sol_*G*^0^/kJ mol^−1^	Δ_sol_*S*^0^/J mol^−1^ K^−1^	*R* ^2^
0.0	14.35	11.91	7.94	0.9995
0.1	13.80	11.31	8.05	0.9956
0.2	13.72	10.67	9.90	0.9980
0.3	13.43	9.98	11.21	0.9975
0.4	13.35	9.25	13.30	0.9988
0.5	13.12	8.59	14.68	0.9992
0.6	12.79	7.94	15.75	0.9980
0.7	12.44	7.28	16.73	0.9984
0.8	12.27	6.65	18.24	0.9989
0.9	11.38	5.99	17.51	0.9976
1.0	11.75	5.32	20.86	0.9967

^b^ Mean relative uncertainties are *u*(Δ_sol_*H*^0^) = 0.07, *u*(Δ_sol_*G*^0^) = 0.25 and *u*(Δ_sol_*S*^0^) = 0.30.

**Table 5:** The parameters of “Van’t Hoff model (*a* and *b*)” along with *R*^2^ and % root mean square deviations (% *RMSD*s) for ECT in various “PEG-400 + water” combinations (*m*) ^c^.

*m*	*a*	*b*	*R* ^2^	*RMSD* (%)	Overall *RMSD* (%)
0.0	0.94	−1724.70	0.9995	0.78	
0.1	0.96	−1657.60	0.9955	0.89	
0.2	1.18	−1648.50	0.9979	0.79	
0.3	1.34	−1614.10	0.9976	0.55	
0.4	1.59	−1604.20	0.9988	0.53	
0.5	1.75	−1575.90	0.9991	0.91	0.73
0.6	1.88	−1537.10	0.9981	0.90	
0.7	2.00	−1494.50	0.9984	0.79	
0.8	2.18	−1474.90	0.9989	0.78	
0.9	2.10	−1368.10	0.9977	0.42	
1.0	2.50	−1412.30	0.9967	0.69	

^c^ The average relative uncertainties are *u*(*a*) = 0.30 and *u*(*b*) = 0.07.

**Table 6:** The parameters of “Apelblat model (*A, B* and *C*)” along with *R*^2^ and % *RMSD*s for ECT in various “PEG-400 + water” combinations (*m*) ^d^.

*m*	*A*	*B*	*C*	*R* ^2^	*RMSD* (%)	Overall *RMSD* (%)
0.0	−81.15	2029.00	12.20	0.9998	0.19	
0.1	−234.90	9119.87	35.05	0.9994	0.67	
0.2	−82.67	2184.81	12.46	0.9985	0.57	
0.3	27.91	−2824.59	−3.95	0.9974	0.62	
0.4	−109.75	3485.30	16.54	0.9996	0.28	
0.5	−97.65	2968.30	14.77	0.9998	0.19	0.46
0.6	−84.07	2392.73	12.77	0.9985	0.48	
0.7	−93.52	2872.33	14.19	0.9990	0.46	
0.8	−61.36	1430.99	9.44	0.9991	0.36	
0.9	24.32	−2380.72	−3.30	0.9975	0.60	
1.0	−56.49	1285.38	8.76	0.9968	0.74	

^d^ The average relative uncertainties are *u*(*A*) = 0.92, *u*(*B*) = 1.54 and *u*(*C*) = 0.90.

**Table 7:** Log *x*^Yal^ values of ECT obtained by the “Yalkowsky-Roseman” model in various “PEG-400 + water” combinations (*m*) at “*T* = 298.2 to 318.2 K”.

*m*	Log *x*^Yal^					*RMSD* (%)	Overall *RMSD* (%)
	298.2 K	303.2 K	308.2 K	313.2 K	318.2 K		
0.1	−1.98	−1.94	−1.90	−1.86	−1.82	2.86	
0.2	−1.87	−1.83	−1.79	−1.75	−1.71	2.98	
0.3	−1.76	−1.72	−1.68	−1.64	−1.60	1.92	
0.4	−1.64	−1.60	−1.57	−1.53	−1.49	0.81	
0.5	−1.53	−1.49	−1.46	−1.42	−1.38	0.87	1.33
0.6	−1.42	−1.38	−1.34	−1.31	−1.27	0.78	
0.7	−1.30	−1.27	−1.23	−1.20	−1.16	0.79	
0.8	−1.19	−1.15	−1.12	−1.09	−1.05	0.57	
0.9	−1.08	−1.04	−1.01	−0.98	−0.94	0.44	

**Table 8:** The parameters of the “Jouyban-Acree” and the “Jouyban-Acree-van’t Hoff” models for ECT in “PEG-400 + water” mixtures.

System	Jouyban-Acree	Jouyban-Acree-van’t Hoff
		*A*_1_ 2.50 *B*_1_ −1412.30 *A*_2_ 0.94 *B*_2_ −1724.70 *J*_i_ 21.32 0.61
PEG-400 + water	*J*_i_ 23.41	
	
	
*RMSD* (%)	0.42	

**Table 9:** List of materials and their properties.

Material	Molecular Formula	Molar Mass (g mol^−1^)	CAS Registry No.	Purification Method	Mass Fraction Purity	Analysis Method	Source
ECT	C_8_H_10_FN_3_O_3_S	247.24	143491-54-7	None	>0.98	HPLC	Sigma-Aldrich
PEG-400	H(OCH_2_CH_2_)_n_OH	400	25322-68-3	None	>0.99	HPLC	Fluka Chemica
Water	H_2_O	18.07	7732-18-5	None	-	-	Milli-Q

The purity and method of analysis was provided by the supplier of each material.
